# A comparison of the biomechanical properties of three different lumbar internal fixation methods in the treatment of lumbosacral spinal tuberculosis: finite element analysis

**DOI:** 10.1038/s41598-023-32624-2

**Published:** 2023-07-13

**Authors:** Jiantao Liu, Xi Gong, Kao Wang, Xingyuan Li, Xiwei Zhang, Jiajun Sun, Yihan Zhu, Yixiang Ai, Jing Ren, Jintao Xiu, Wenchen Ji

**Affiliations:** 1 Department of Orthopedics, First Affiliated Hospital of Xi’an Jiaotong University, Xi’an, 710061 China; 2 Xi’an Jiaotong University, Xi’an, 710049 China; 3 Medical School of Yan’an University, Yan’an, 716000 China

**Keywords:** Biophysical methods, Biomedical materials

## Abstract

There are various internal fixation methods in treating lumbosacral spinal tuberculosis. The study compared the stability and stress distribution in surrounding tissues/implants, such as discs, endplates and screw-rod internal fixation system, etc. when applying three different lumbar internal fixation methods to treat lumbosacral spinal tuberculosis. A finite element model was constructed and validated. The spinal stability was restored using three methods: a titanium cage with lateral double screw-rod fixation (group 1), autologous bone with posterior double screw-rod fixation (group 2), and a titanium cage with posterior double screw-rod fixation (group 3). For comparison, group 4 represented the intact L3-S1 spine. Finally, a load was applied, and the ranges of motion and Von Mises stresses in the cortical endplates, screw-rod internal fixation system and cortical bone around the screws in the different groups were recorded and analyzed. All six ranges of motion (flexion, extension, left/right lateral bending, left/right rotation) of the surgical segment were substantially lower in groups 1 (0.53° ~ 1.41°), 2 (0.68° ~ 1.54°) and 3 (0.55° ~ 0.64°) than in group 4 (4.48° ~ 10.12°). The maximum stress in the screw-rod internal fixation system was clearly higher in group 2 than in groups 1 and 3 under flexion, left/right lateral bending, and left/right rotation. However, in extension, group 1 had the highest maximum stress in the screw-rod internal fixation system. Group 2 had the lowest peak stresses in the cortical endplates in all directions. The peak stresses in the cortical bone around the screws were higher in group 1 and group 2 than in group 3 in all directions. Thus, titanium cage with posterior double screw-rod fixation has more advantages in immediate reconstruction of lumbosacral spinal stability and prevention of screw loosening.

## Introduction

Spinal tuberculosis, especially in the thoracic and lumbar segments, is a common type of extrapulmonary tuberculosis that accounts for approximately 50% of osteoarticular tuberculosis cases^1–3^. With the increase in the number of patients with acquired immunodeficiency and the widespread use of immunosuppressive drugs, the number of spinal tuberculosis patients is also increasing annually^4,5^. Delayed treatment of spinal tuberculosis may lead to vertebral bone destruction and collapse, kyphosis and even paralysis due to compression of the spinal cord or nerves^6–8^. Currently, drug therapy consisting of isoniazid, rifampicin, ethambutol, and pyrazinamide is the preferred treatment for spinal tuberculosis. However, drug therapy cannot easily cure “closed lesions.” Moreover, 8% to 12% of patients have resistance to first-line antitubercular drugs^4^. Surgical treatment is still necessary for patients with spinal cord injury and spinal instability due to tuberculosis lesions^9,10^. The purpose of such operations is to completely remove the lesions, correct spinal deformities, relieve nerve compression, and improve the patient’s quality of life. Three main surgical strategies for treating lumbosacral spinal tuberculosis are the anterior-only approach, the posterior-only approach, and the combined anterior and posterior approach^11^. Although each approach has its own advantages and disadvantages, all three approaches have been widely used in clinics and have achieved satisfactory clinical efficacy for patients with lumbosacral spinal tuberculosis. Regardless of the surgical approach, the three most common internal fixation methods for restoring spinal stability after lesion removal are a titanium cage combined with lateral double screw-rod fixation^12^, autologous bone combined with posterior double screw-rod fixation^13^, and a titanium cage combined with posterior double screw-rod fixation^14^. However, clinical follow-up showed that the three internal fixation methods had complications such as prosthesis subsidence, screw loosening and titanium rod fracture, etc., and there is still a lack of relevant research on which internal bone fixation method has more advantages in mechanical properties. Therefore, to evaluate the influence of the three different internal fixation methods on the stability of the lumbosacral segment and the surrounding tissues during spinal reconstruction, we performed finite element (FE) analyses and compared the biomechanical properties of the three methods. The ranges of motion (ROMs) and Von Mises stresses in the discs, facet joints, cortical endplates, screw-rod internal fixation system and cortical bone around the screws in the different groups were compared. The results of this study will provide a useful reference for the clinical application of the three different internal fixation methods.

## Methods and materials

### FE modelling of the intact L3-S1 spine

According to the modelling processes reported by some previous studies^15–18^, a healthy adult male volunteer (31 years old, 175 cm, 68 kg) underwent a CT scan (0.625 mm, GE Lightspeed VCT-XT 64) of the L3-S1 spine. Then, the CT scan data were imported into Mimics (Version 20.0, Materialise Inc., Leuven, Belgium) to generate a surface model of the vertebrae. In addition, 3-matic software (Materialise Inc.) was used to construct solid models of the cortical shell (1125 Hounsfield Unit), cancellous bone (220 Hounsfield Unit), and intervertebral disc. Meshed models of the bony and ligamentous structures were constructed using Hypermesh (Altair Engineering, Inc., Troy, Michigan, USA). Abaqus (Hibbitt, Karlsson, and Sorensen, Inc., Providence, Rhode Island, USA) was used for material property definitions, model assembly, and FE analysis. Mesh refinement was executed for modeling accuracy until excellent monotonic convergence behavior with less than 5% difference in the L3-top displacement was achieved In this study, the boundary conditions described below were applied to the models, and convergence was tested for based on range of motion, bone strain, and disc stress. The size and density of the hexahedral elements was controlled manually, while the density of the tetrahedral elements was controlled by determining an appropriate average element side length.

**Figure 1:**
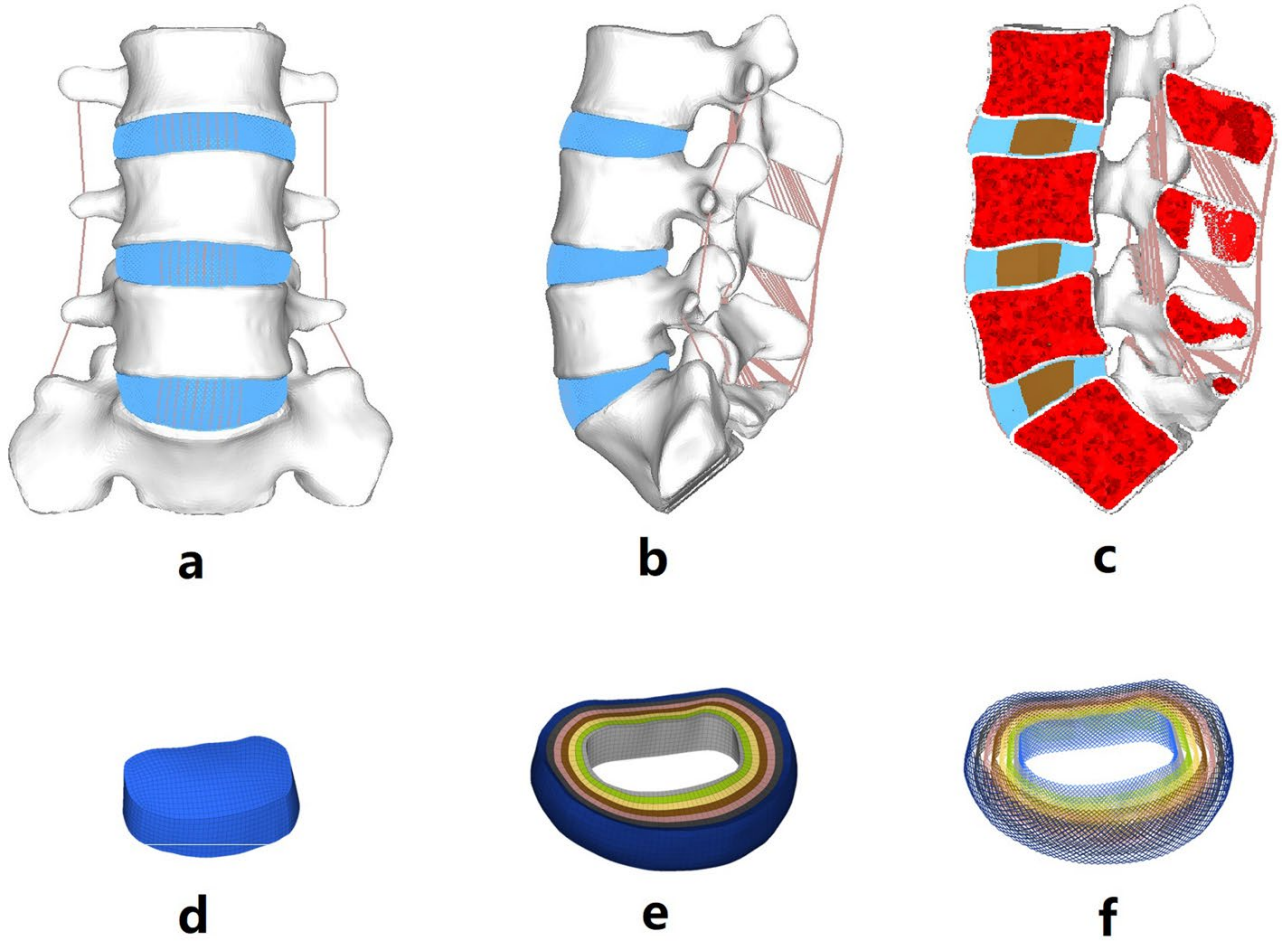
FE model of the intact lumbosacral spine. (**a**) the front view of the intact FE model, (**b**) the lateral view of the intact FE model, (**c**) the sagittal sectional view of the intact FE model, (**d**) FE model of the nucleus pulposus, (**e**) FE model of the annulus ground substance, (**f**) FE model of the annulus fibrosus.

Figure 1 shows an FE model of the lumbosacral spine. The thickness of the cortical shell and cartilage endplate was 1 mm^19,20^. The intervertebral disc was divided into the nucleus pulposus and annulus fibrosus. The nucleus pulposus accounts for 30 ~ 40% of the intervertebral volume^20^. The nucleus pulposus was modelled as a linearly elastic solid element. The annulus fibrosus consisted of the annulus ground substance and fibres. Seven layers of annulus fibres were embedded into the annulus ground substance at an inclination of ± 30°^19,20^. The elastic modulus of the annulus fibres proportionally increased and varied from the innermost layer (360 MPa) to the outermost layer (550 MPa)^21^. In total, 7 ligaments were modelled: the anterior longitudinal ligament, posterior longitudinal ligament, ligamentum flavum, capsular ligament, interspinous ligament, supraspinous ligament, and intertransverse ligament. The element types and material properties used in the FE model (Table 1) were defined in accordance with previous reports^15,22–25^. For the intact model is consist of 1,005,661 elements connected through 333,880 nodes. To validate the intact L3-S1 FE model, the intervertebral (segmental) ranges of motion (ROMs) in response to 8.0 Nm loads were compared with the outcomes described in previous publications^23^.

**Table 1:** Material properties assigned to the Fe model.

Component element	Type	Young modulus (MPa)	Poisson ratio	Cross-sectional area (mm^2^)
Cortical bone	C3D4	12,000	0.3	–
Cancellous bone	C3D4	100	0.2	–
Articular cartilage	C3D4	10.4	0.4	–
Nucleus pulpous	C3D8H	1	0.49	–
Annulus fibers	T3D2	360 ~ 550	0.3	–
Annulus ground substance	C3D8H	3.4	0.4	–
Anterior longitudinal ligament	T3D2	7.8(< 12%), 20(> 12%)		6.37
Posterior longitudinal ligament	T3D2	10(< 11%), 20(> 11%)		2
Ligamentum flavum	T3D2	15(< 6.2%), 19.5(> 6.2%)		4
Capsular ligament	T3D2	7.5(< 25%), 32.9(> 25%)		5.5
Interspinous ligament	T3D2	10(< 20%), 11.6(> 20%)		4
Intertransverse ligament	T3D2	10(< 18%), 58.7(> 18%)		1
Supraspinous ligament	T3D2	8(< 20%), 15(> 20%)		10

### FE modelling of the three different lumbar internal fixation methods

**Figure 2:**
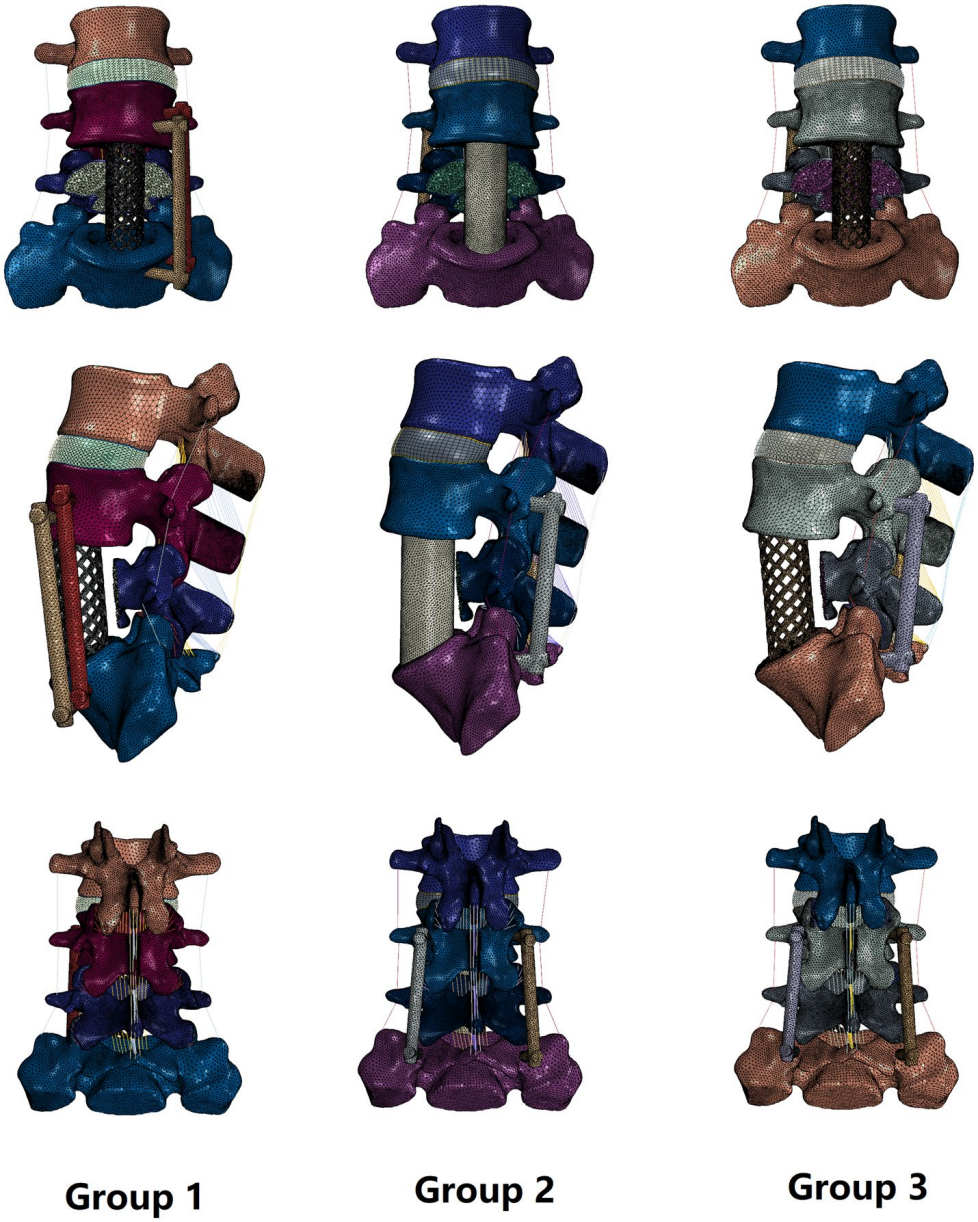
FE models of three lumbar internal fixation methods. Group 1-titanium cage combined with lateral double screw-rods fixation; Group 2-autologous bone combined with posterior double screw-rod fixation; Group 3-titanium cage combined with posterior double screw-rods fixation.

The FE models of the three lumbar internal fixation methods are shown in Fig. 2. To simulate the surgical procedure in the treatment of lumbosacral tuberculosis, the L5 vertebra and adjacent discs (L4-5 disc and L5-S1 disc) and the corresponding anterior and posterior longitudinal ligaments were removed, while the other structures, including the posterior bony elements, ligamentum flavum, and capsular ligament, were preserved. After decompression, three different internal fixation methods were used to restore the stability of the surgical site: a titanium cage combined with lateral double screw-rod fixation (group 1), autologous bone combined with posterior double screw-rod fixation (group 2), and a titanium cage combined with posterior double screw-rod fixation (group 3). For comparison, we set the FE model of the intact L3-S1 spine as group 4. Four screws and two rods were used in all three internal fixation methods. The length and diameter of the screws were 45 mm and 6.5 mm, respectively, and the diameter of the titanium rods was 5.5 mm. Four screws were placed in the adjacent vertebrae (L4 and S1 vertebrae). In group 1, four screws were implanted along the lateral side of the vertebrae, whereas the screws were implanted along the vertebral pedicles in group 2 and group 3. In contrast to the vertebral lesions, the titanium cage and autogenous bone are cylinders of the same size. Geometric matching at the prosthesis-endplate interface was achieved using “Boolean calculations” to remove the portion of the cage and autogenous bone that overlapped with the vertebral body. The FE models in groups 1, 2, and 3 separately have 929,690, 872,580, 871,893 elements and are connected by 196,991, 188,849, 189,209 nodes. The element types and material properties of the different implants were also defined based on data in previous publications (Table 2)^25^. For all surgical models, the cage-end plate and screw-bone interfaces were defined as tied contacts to simulate internal fixation. See Table 3 for contact state settings.

**Table 2:** Material properties assigned to different prostheses.

Component element	Type	Young modulus (MPa)	Poisson ratio	Cross-sectional area (mm^2^)
Autogenous bone	C3D4	100	0.2	–
Titanium alloy	C3D4	110,000	0.28	–

**Table 3:** Contact state setting list of each contact part in the finite element model.

Number	Contact pairs	Contact state setting
1	Reference point and vertebrae	Coupling
2	Facet joint	Surface to surface with frictionless
3	Annulus fibers and annulus ground substance	Embedded
4	Ligaments and vertebrae	Embedded
5	New prosthesis, titanium cage and vertebrae	Tie

The reference point refers to the origin of our coordinate system in finite element analysis.

### Loading and boundary conditions

**Figure 3:**
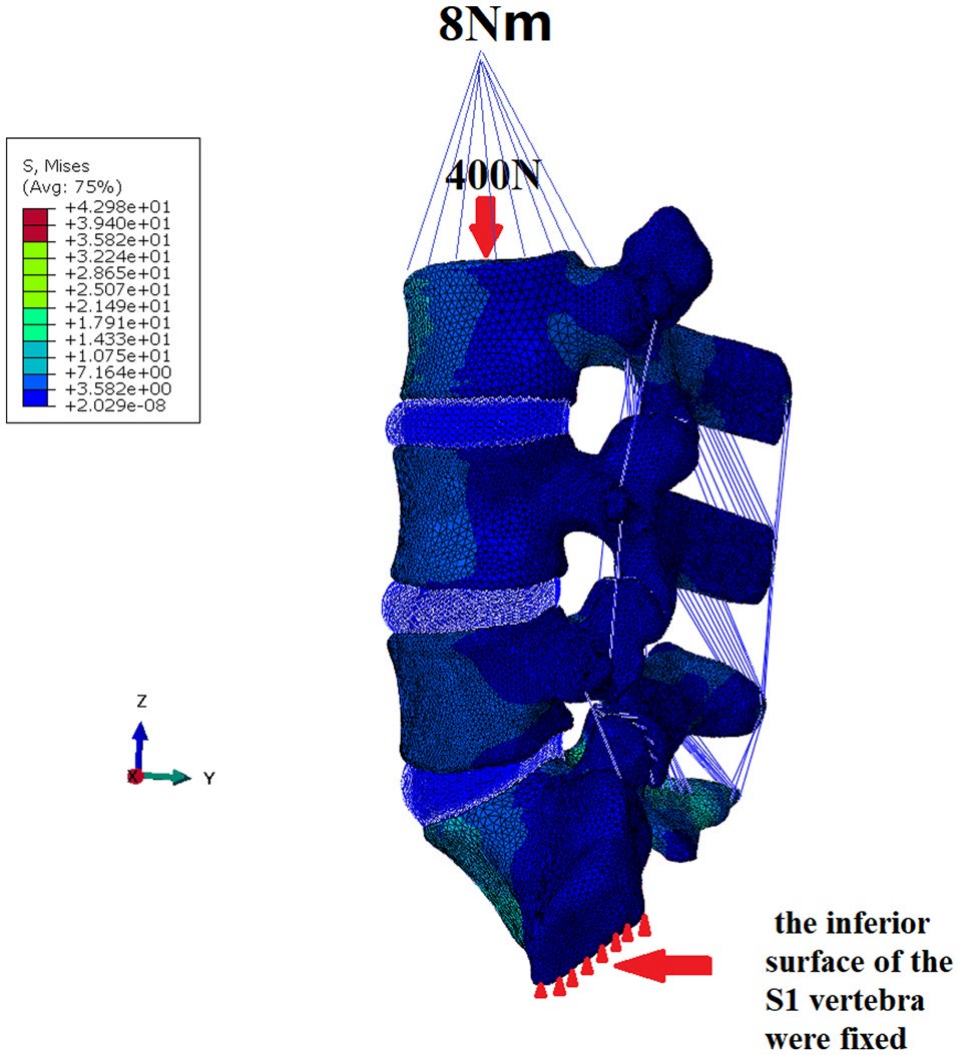
Schematic diagram of loading and boundary conditions.

As shown in Fig. 3, for all FE models, all degrees of freedom of the inferior surface of the S1 vertebra were fixed, and two kinds of simplified loads were applied to the superior surface of the L3 vertebra. One type of load is the Preload (Follow load), 400N, transmitted along the physiological curve of the spine, and the other type of load is the pure bending moment (8 Nm), which causes the spine to move in flexion (FLX), extension (EXT), left lateral bending (LLB), right lateral bending (RLB), left rotation (LT) and right rotation (RT). The combined effect of both is the effect of muscle mass and gravity. The ROMs and maximum Von Mises stresses in the internal fixation system, the endplate and the bone around the screws of FE models were analysed under an 8 Nm load.

### Ethical approval

This study was carried out in accordance with the Code of Ethics of the World Medical Association (Declaration of Helsinki) and approved by the Ethics Committee of Xi’an Jiaotong University. A computed tomography (CT) scan of the L3-S1 spine was obtained with the informed consent of volunteers, and the dissemination of relevant data was allowed for academic exchange.

## Results

### Model validation

**Figure 4:**
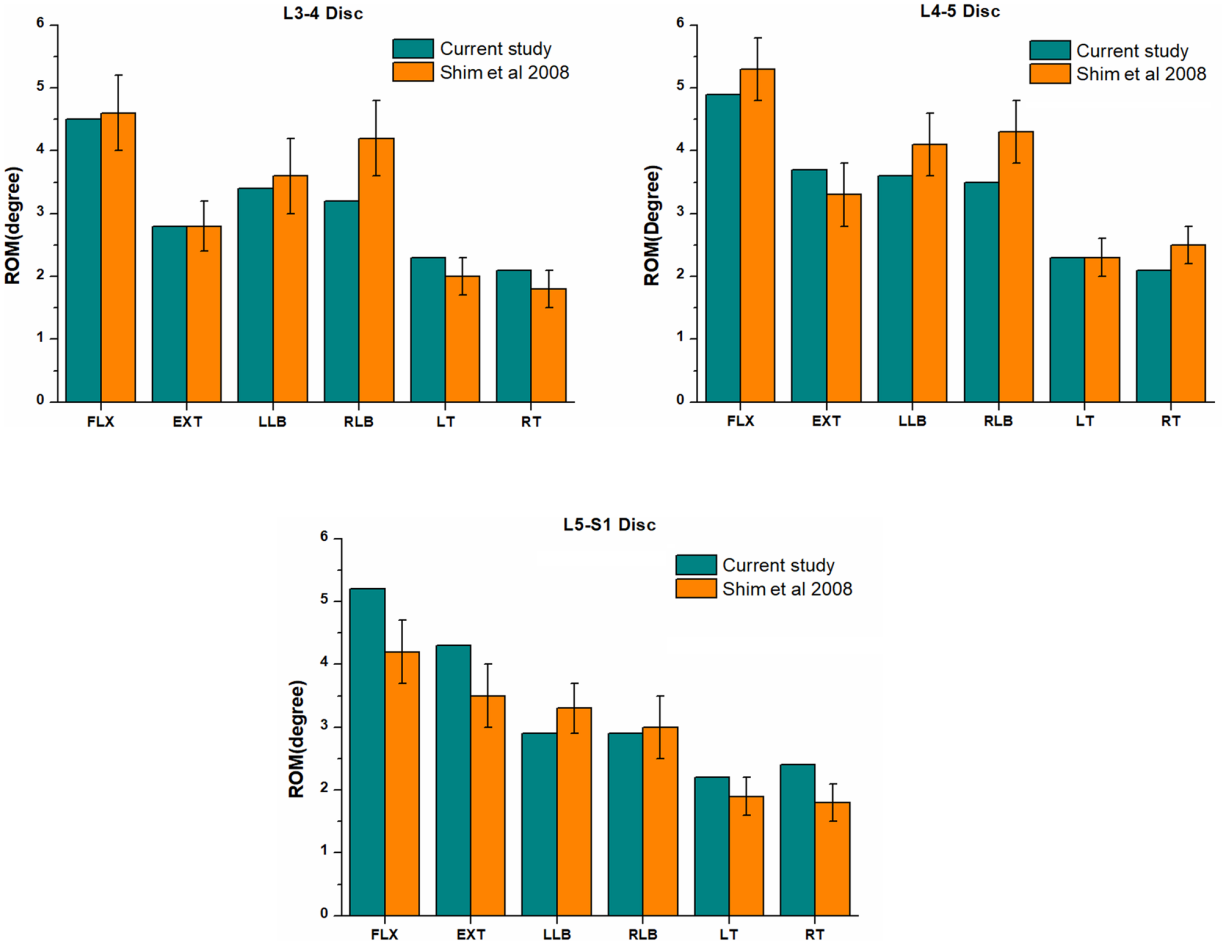
Comparison between the predicted L3-S1 ROMs (under moments of 8 Nm) and those obtained in with the relevant literature.

**Figure 5:**
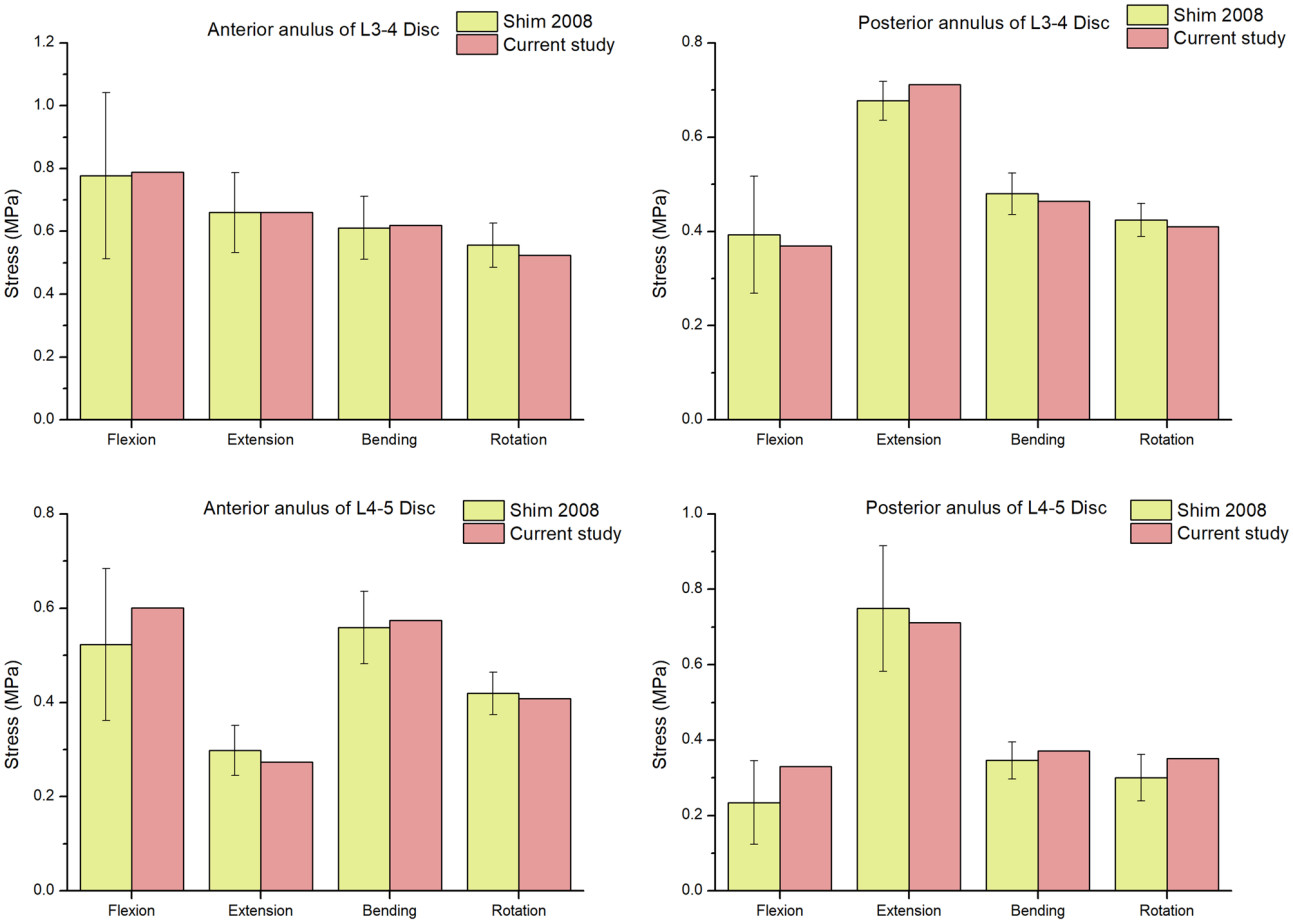
Comparison between the stresses of L3–4 and L4–5 disc in the current study and those obtained in Shim’s previous biomechanical studies.

Figure 4 shows a comparison between the L3-S1 ROMs (under a moment of 8 Nm) of the intact FE model and those obtained in the previous studies^26^. The intersegmental ROMs of the intact FE model at the L3-4 disc in the FLX, EXT, LLB, RLB, LT and RT directions were 4.5°, 2.8°, 3.4°, 3.2°, 2.3° and 2.1°, respectively. In contrast, the ROMs of the L4-5 disc in these directions were 4.9°, 3.7°, 3.6°, 3.5°, 2.3° and 2.1°, respectively. Moreover, the ROMs of the L5-S1 disc in these directions were 5.2°, 4.3°, 2.9°, 2.9°, 2.2° and 2.4°, respectively. Compared with the results of Shim's study^26^, the ROM of the L3-4 disc in our study was reduced by 9.5% in the RLB direction; the ROMs of the L4-5 disc in RLB and RT directions decreased by 7.0% and 4.0%, respectively; the ROMs of the L5-S1 disc in FLX, EXT and RT directions increased by 10.6%, 7.5% and 14.3%, respectively. However, The ROM results of the discs in our study in other directions were in agreement with the findings of Shim’s study. In addition, we compared the stress of the intervertebral disc of L3-4 and L4-5 with the relevant literature (Fig. 5), and the results also showed that the stress of the intervertebral disc constructed by the model was consistent with the results of in vitro mechanical test reported in the literature^26^ . Based on the above verification results, the intact L3-S1 FE model in the present study was successfully constructed and could be used for further analysis.

### ROMs of surgical segments

**Figure 6:**
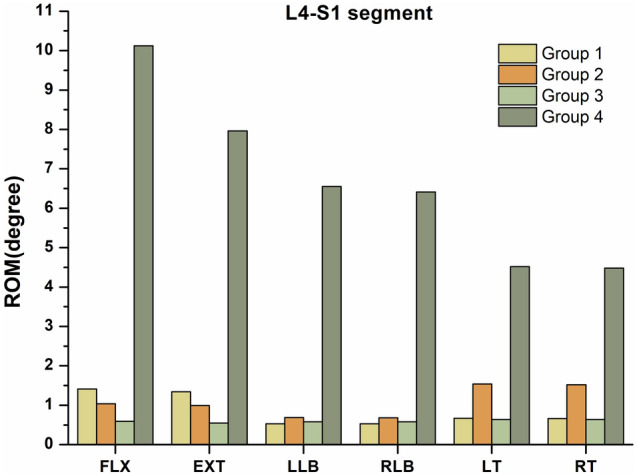
Comparisons of the ROMs observed at surgical segments (L4-S1) among the four groups. Group 1—titanium cage combined with lateral double screw-rods fixation; Group 2—autologous bone combined with posterior double screw-rod fixation; Group 3—titanium cage combined with posterior double screw-rods fixation; Group 4—the intact FE model.

A total of three internal fixation methods were modelled and analysed, and the ROMs of the surgical segments (L4-S1) in the FLX, EXT, LLB, RLB, LT and RT directions are shown in Table 4. A comparison of the ROMs of the L4-S1 segments among the three different internal fixation methods is shown in Fig. 6. According to the results, the ROMs of L4-S1 in group 1, group 2 and group 3 were 0.53° ~ 1.41°, 0.68° ~ 1.54°, and 0.55° ~ 0.64° less than those in group 4 (4.48° ~ 10.12°), respectively. The ROMs of L4-S1 in all six directions in group 3 were substantially lower than those in group 2, whereas the ROMs of L4-S1 in the LLB, RLB, LT and RT directions in group 1 were similar to those in group 3. However, the ROMs of L4-S1 in the FLX and EXT directions in group 1 were substantially larger than those in groups 2 and 3.

**Table 4:** The ROMs at surgical segments (L4-S1) among the four groups (degree).

Group	FLX	EXT	LLB	RLB	LT	RT
L4-S1						
Group 1	1.41	1.34	0.53	0.53	0.67	0.66
Group 2	1.04	0.99	0.69	0.68	1.54	1.52
Group 3	0.59	0.55	0.58	0.58	0.64	0.64
Group 4	10.12	7.96	6.55	6.41	4.52	4.48

FLX, flexion, EXT, extension, LLB, left lateral bending, RLB, right lateral bending, LT, left rotation, RT, right rotation. Group 1—titanium cage combined with lateral double screw-rods fixation; Group 2—autologous bone combined with posterior double screw-rod fixation; Group 3—titanium cage combined with posterior double screw-rods fixation; Group 4—the intact L3-S1 spine.

### Internal fixation system stress

**Figure 7:**
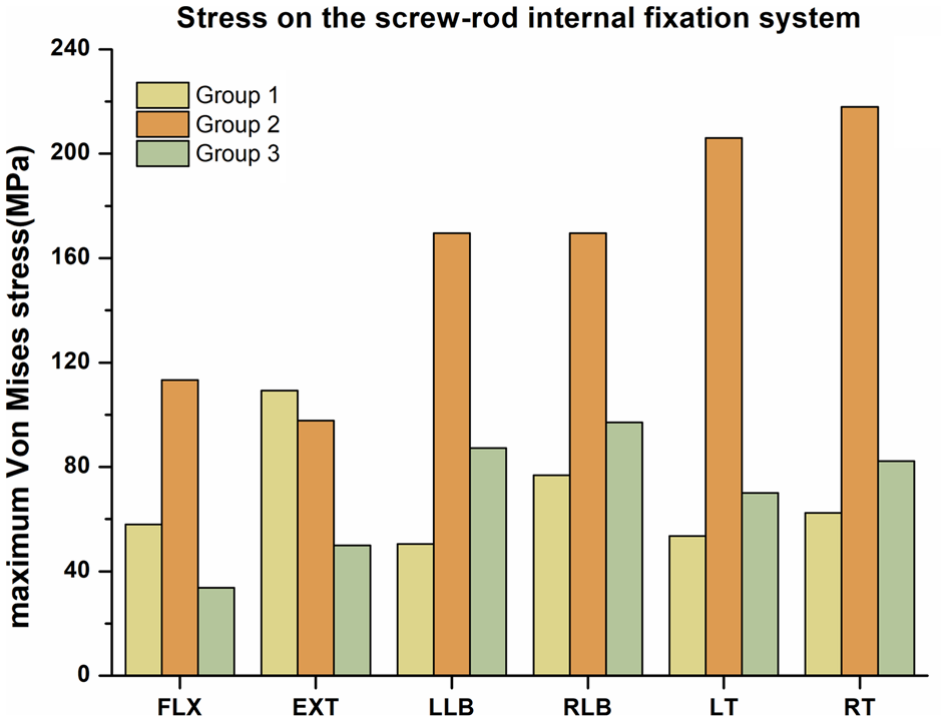
The maximum Von Mises stresses on the screw-rod fixation system in the three groups.

**Figure 8:**
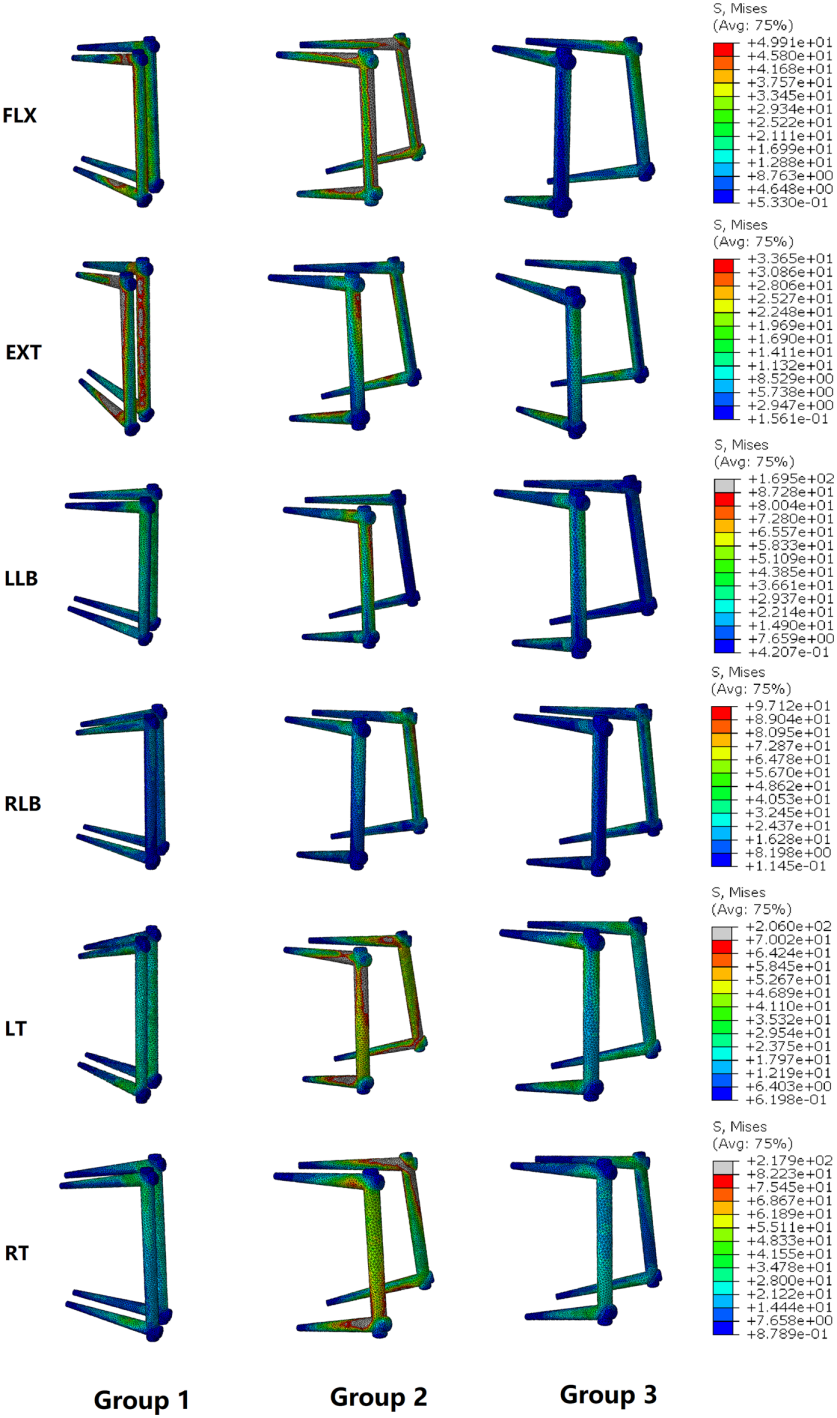
The distributions of Von Mises stresses on the screw-rod fixation system in the three groups during FLX, EXT, LLB, RLB, LT and RT.

Figure 7 shows a comparison of the maximum Von Mises stress in the screw-rod internal fixation system in all six directions in the three groups. According to the results, in the FLX direction, the maximum Von Mises stress in the screw-rod internal fixation system in group 2 was 113.3 MPa, which was substantially larger than that in group 1 (58.0 MPa) and group 3 (33.7 MPa). However, the maximum Von Mises stress in the screw-rod internal fixation system in group 1 in the EXT direction was 109.3 MPa, followed by that in group 2 (97.8 MPa) and group 3 (49.9 MPa). In the LLB, RLB, LT and RT directions, the maximum Von Mises stresses in the screw-rod internal fixation system in group 2 were 169.5, 169.5, 206.0 and 217.9 MPa, respectively, which were substantially higher than those in group 1 (50.6, 76.8, 53.5, and 62.4 MPa, respectively) and group 3 (87.3, 97.1, 70.0, and 82.2 MPa, respectively). The maximum Von Mises stresses in the screw-rod internal fixation system in group 1 in the LLB, RLB, LT and RT directions were 16.5 ~ 36.7 MPa less than those in group 3. The stress distributions in the screw-rod internal fixation system in the six directions above in the three groups are shown in Fig. 8. The maximum Von Mises stresses in the internal fixation system in the three groups were all located at the screw-rod joint.

### Cortical endplate stress

**Figure 9:**
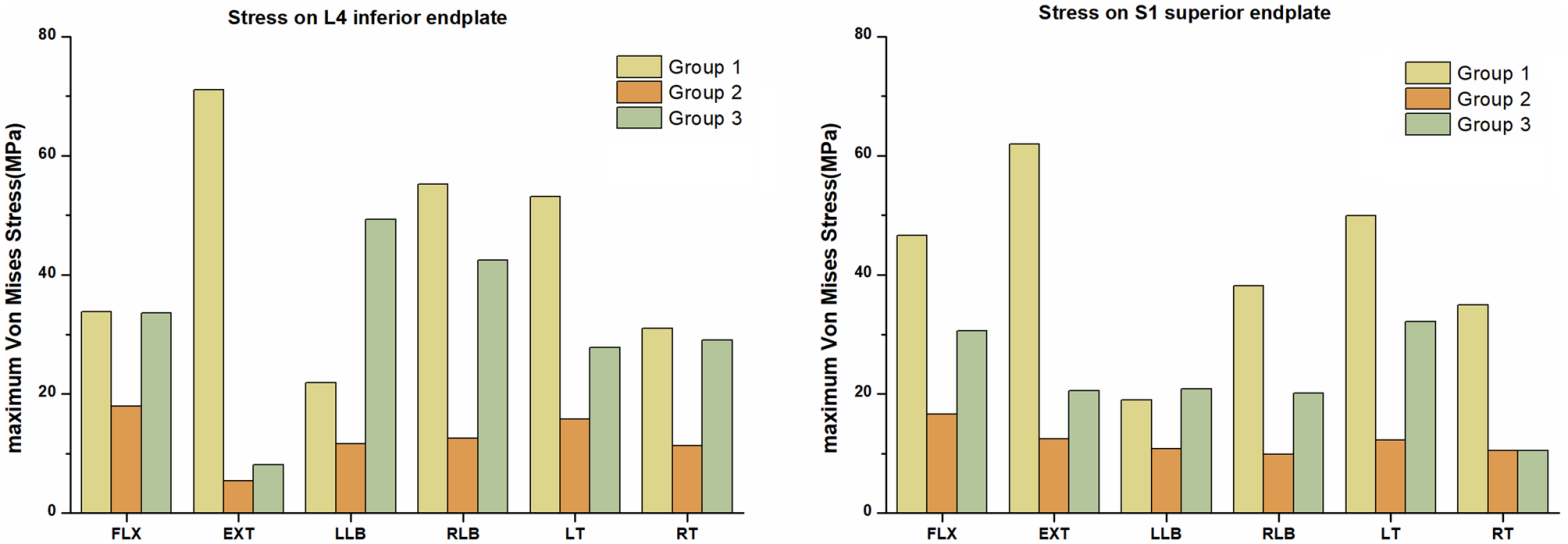
Comparisons of the maximum Von Mises stresses on the L4 inferior endplates and S1 superior endplates in the three groups.

**Figure 10:**
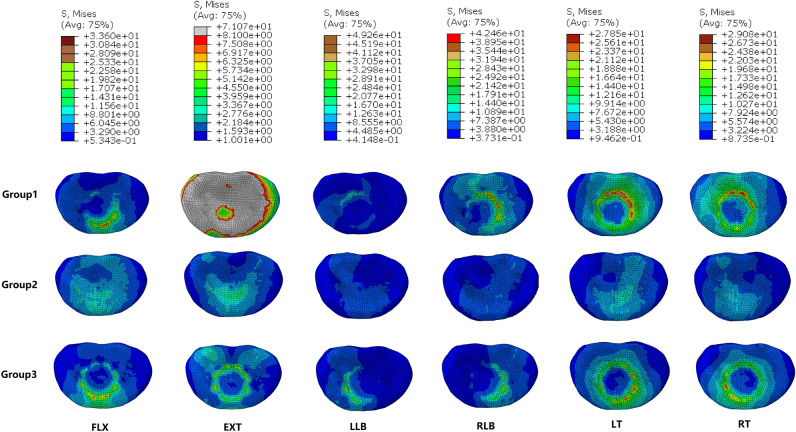
The distributions of Von Mises stresses on the L4 inferior endplate in the three groups.

Figure 9 and Table 5 show the maximum Von Mises stresses recorded at the cortical endplates. According to the results, for all six direction, the lowest peak stresses in the cortical endplates were in group 2. The peak stresses in the cortical endplates in the FLX, EXT, RLB, LT and RT directions were highest in group 1, followed by those in group 3, for which the stresses at the L4 inferior endplate and S1 superior endplate were reduced by 0.2 ~ 63.0 MPa and 16.0 ~ 41.3 MPa, respectively. However, the maximum Von Mises stresses in the cortical endplates in group 1 were lower than those in group 3 in the LLB direction, wherein the stresses at the L4 inferior endplate and S1 superior endplate were reduced by 27.3 MPa and 1.9 MPa, respectively. Figure 10 shows the stress distributions in the L4 inferior endplates.

**Table 5:** The maximum Von Mises stresses at the adjacent endplates in the three groups (MPa).

Group	FLX	EXT	LLB	RLB	LT	RT
L4 inferior endplate						
Group 1	33.8	71.1	21.9	55.2	53.1	31.1
Group 2	17.9	5.5	11.7	12.5	15.8	11.3
Group 3	33.6	8.1	49.3	42.5	27.9	29.1
S1 superior endplate						
Group 1	46.6	61.9	19.0	38.2	50.0	35.0
Group 2	16.7	12.5	10.8	9.9	12.3	10.5
Group 3	30.6	20.6	20.9	20.1	32.2	10.5

FLX, flexion, EXT, extension, LLB, left lateral bending, RLB, right lateral bending, LT, left rotation, RT, right rotation. Group 1—titanium cage combined with lateral double screw-rods fixation; Group 2—autologous bone combined with posterior double screw-rod fixation; Group 3—titanium cage combined with posterior double screw-rods fixation.

### Stress in the cortical bone around the screws

**Figure 11:**
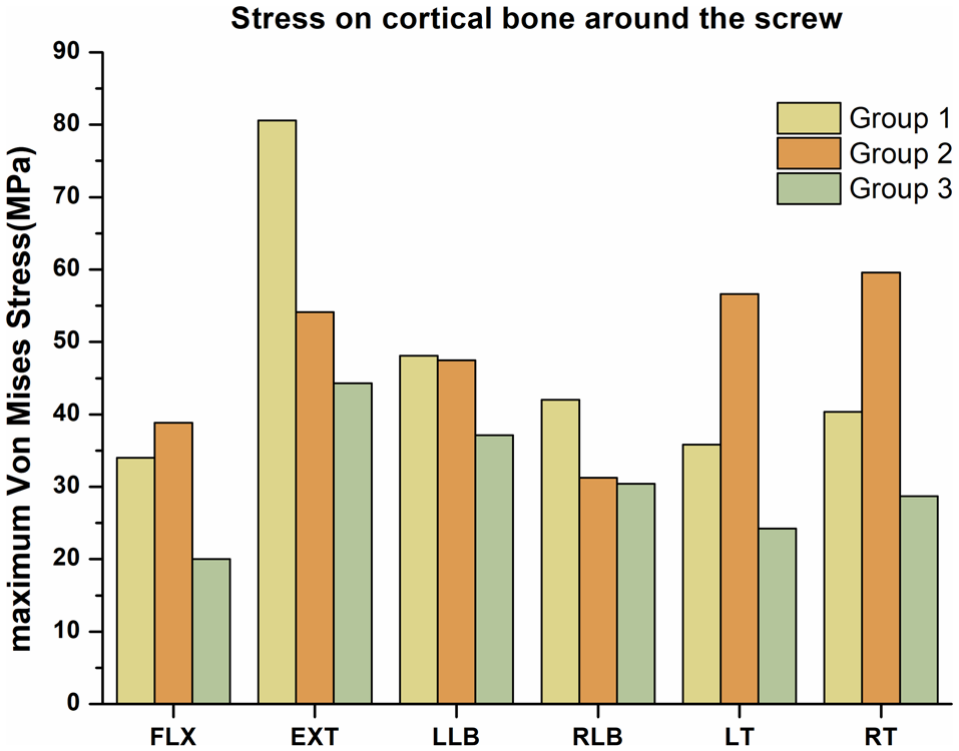
Comparisons of the maximum Von Mises stresses on the cortical bone around the screw in the three groups.

Figure 11 shows a comparison of the maximum Von Mises stress in the cortical bone around the screws in the three groups. According to the results, the maximum Von Mises stresses in the cortical bone around the screws in the FLX, EXT, LLB, RLB, LT and RT directions were lowest in group 3, for which the values were 20.0 MPa, 44.3 MPa, 37.1 MPa, 30.4 MPa, 24.2 MPa and 28.7 MPa, respectively. In the FLX, LT and RT directions, the maximum Von Mises stresses in the cortical bone around the screws in group 2 (38.8 MPa, 56.6 MPa, and 59.6 MPa, respectively) were larger than those in group 1 (34.0 MPa, 35.8 MPa, and 40.3 MPa, respectively). In contrast, in the EXT, LLB and RLB directions, the Von Mises stresses in the cortical bone around the screws in group 2 (54.1 MPa, 47.5 MPa, and 31.2 MPa, respectively) were lower than those in group 1 (80.6 MPa, 48.1 MPa, and 42.0 MPa, respectively).

### Stress on discs and facet joints

**Figure 12:**
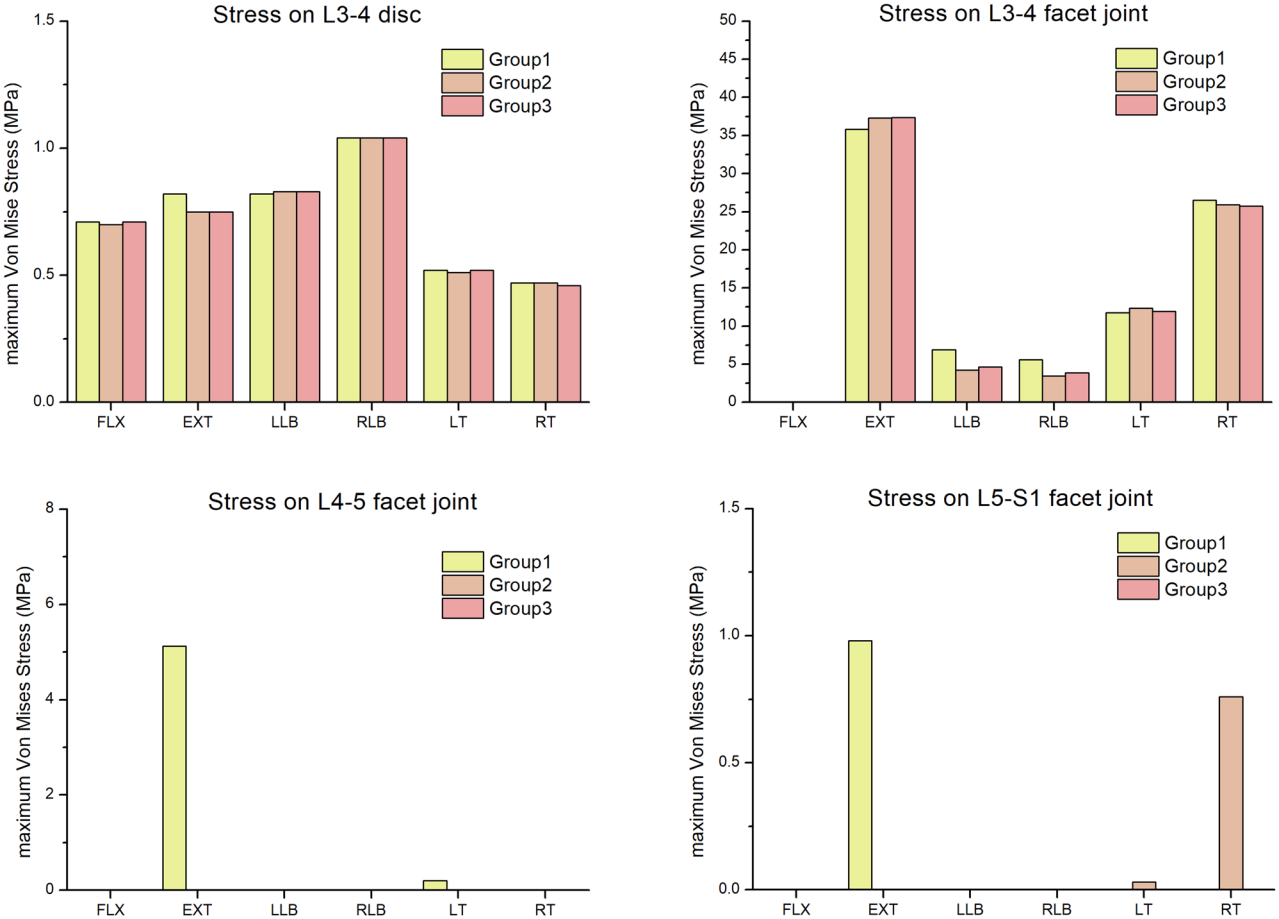
Comparisons of the maximum Von Mises stresses on the L3–4 disc and facet joints in the three groups.

**Figure 13:**
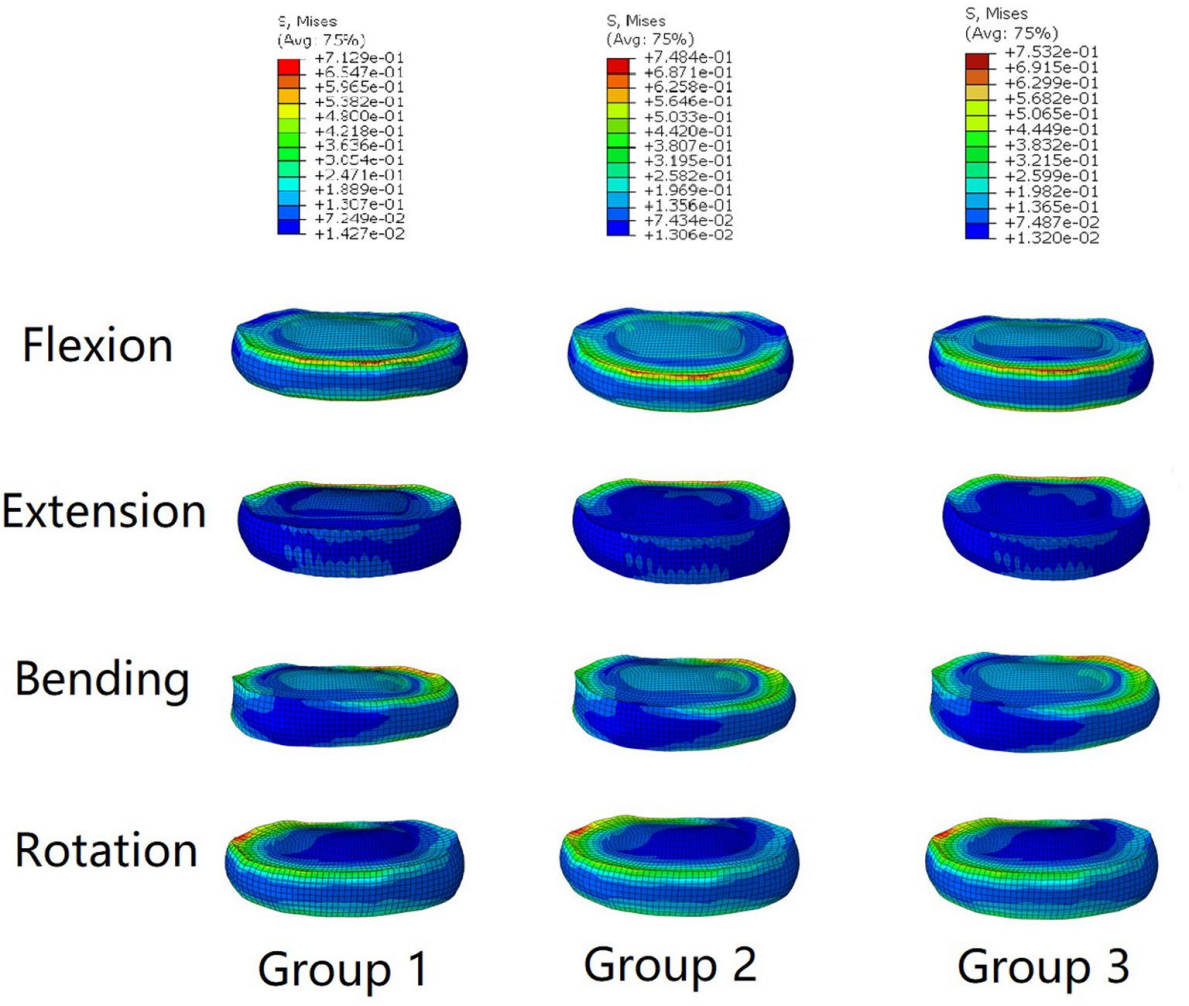
The distributions of Von Mises stresses on the L3–4 disc in the three groups.

The comparisons of the maximum Von Mises stresses of the L3-4 disc and facet joints of the three groups were shown in Fig. 12. According to the results, the maximum Von Mises stress of L3-4 disc in the three groups was almost the same in the direction of FLX, LLB, RLB, LT and RT. However, in the EXT direction, the maximum Von Mises stress of the L3-4 in group 1(0.82 MPa) slightly higher than that in group 2 ~ 3(0.75 MPa). The distributions of Von Mises stresses on the L3-4 disc in the three groups were shown in Fig. 13. The maximum Von Mises stress of L3-4 facet joints in the three groups had a small difference in the direction of FLX, LT and RT. The maximum Von Mises stress of L3-4 facet joints in group 1(35.81 MPa) was slightly lower than that in groups 2(37.32 MPa) and 3(37.38 MPa) in the EXT direction, while in the LLB and RLB direction, the stress in group 1(6.92 MPa, 5.61 MPa) was slightly higher than that in group 2(4.22 MPa, 3.44 MPa) and group 3(4.62 MPa, 3.88 MPa). The maximum Von Mises stresses of L4-5 and L5-S1 facet joints in group 3 was 0 in the directions of FLX, EXT, LLB, RLB, LT and RT. The maximum Von Mises stress of L4-5 facet joints in group 2 was also 0 in the above six directions, while the maximum Von Mises stress of L5-S1 facet joints of group 2 in the LT and RT directions was 0.03 MPa and 0.76 MPa, respectively. The maximum Von Mises stress of L4-5 and L5-S1 facet joints of group 1 in the EXT direction was 5.12 MPa and 0.98 MPa, respectively, while the maximum Von Mises stress of L4-5 facet joints of group 1 in the LT direction was 0.2 MPa, and the stresses in other directions were 0.

## Discussion

Lumbosacral spinal tuberculosis accounts for 2–3% of all cases of spinal tuberculosis^27^. Surgical interventions are required for patients with progressive neurological functional impairment, severe kyphosis, and massive cold abscesses and for those who have had no response to conservative treatment^28^. The three most common surgical strategies used for patients with spinal tuberculosis are a titanium cage combined with lateral double screw-rod fixation, autologous bone combined with posterior double screw-rod fixation, and a titanium cage combined with posterior double screw-rod fixation. However, it is not clear which internal fixation method can best restore the stability of the surgical site and reduce the incidence of prosthesis loosening and displacement. In this study, FE analyses were performed to compare the biomechanical properties of these three internal fixation methods, the results of which should provide a useful reference for their clinical application.

This study showed that the ROMs of L4-S1 in the three internal fixation groups (groups 1–3) were substantially lower than those in group 4, which was consistent with the reduced ROMs at the surgical site after internal fixation^10,12,29^, suggesting that all three methods of internal fixation can restore the stability of the surgical site after lesion removal. Further analysis showed that the ROMs of L4-S1 in group 3 were lower than those in group 2, indicating that under posterior double screw-rod fixation, a titanium cage could achieve better stability than autologous bone. Wu W et al.^14^ also compared titanium cage with autograft in the treatment of spinal tuberculosis, and found that titanium cage combined with posterior screw-rod fixation was more advantageous, which is consistent with our findings. Although group 1 and group 3 had similar ROMs at the surgical site in the LLB, RLB, LT and RT directions, the ROMs of L4-S1 in group 1 were higher than those in group 3 in the FLX and EXT directions, indicating that after titanium cage implantation, posterior double screw-rod fixation could achieve better stability in the FLX and EXT directions than lateral double screw-rod fixation. Reisener et al.^30^ reviewed the literature and found that titanium cage combined with posterior screw- rod fixation was more common than titanium cage combined with lateral double screw-rod fixation in the treatment of lumbosacral spinal diseases. In addition, Zheng et al.^10^ found that posterior screw-rod fixation was more advantageous than lateral double screw-rod fixation in rebuilding the stability of the lumbosacral spine. Thus, a titanium cage combined with posterior double screw-rod fixation can best restore the stability of the surgical site.

In all six directions, the maximum Von Mises stresses in the screw-rod internal fixation system in group 3 were substantially lower than those in group 2. This indicates that compared to anterior autologous bone implantation, anterior titanium cage implantation can reduce the stress in the posterior double screw-rod system and can reduce the risk of fracture of the screw-rod system to a certain extent. This is consistent with the research results of Wu et al.^14^. However, the maximum Von Mises stresses in the screw-rod system in group 1 were higher in the FLX and EXT directions and lower in the LLB, RLB, LT and RT directions than those in group 3. This indicates that the two internal fixation methods have their own advantages in different movement directions. As lumbar FLX and EXT occur much more frequently in daily life than LLB/RLB and LT/RT, a titanium cage combined with posterior double screw-rod fixation seems to have more relevant advantages. Bian et al.^11^ also found through systematic review that the incidence of implant failure in posterior fixation was lower than that in anterior fixation, which is consistent with our findings.

In this study, group 2 had the lowest peak Von Mises stresses in the cortical endplates in all six directions. One possible reason is that autologous bone has a larger contact area than titanium cage, which can better disperse the stress to a certain extent. The other reason may be that autogenous bone has a lower elastic modulus than a titanium cage and has a certain buffering function under load, which can reduce the pressure on the endplate to a certain extent. The lower the cortical endplate stresses are, the lower the frequency of implant subsidence in the future. Therefore, autologous bone grafting is more advantageous in preventing subsidence of the prosthesis. Wang et al.^31^ also found that traditional titanium cages had defects of high subsidence rate, which was closely related to high local stress, which also confirmed our research results. The maximum Von Mises stresses in the cortical endplates in group 1 were higher than those in group 3 in all directions of motion except the LLB direction. This indicates that after anterior titanium cage implantation, posterior double screw-rod fixation provides a greater stress reduction in the adjacent cortical endplates than lateral double screw-rod fixation, thereby reducing the incidence of titanium cage subsidence. Although the clinical follow-up study of Zhang et al.^12^ did not find obvious prosthesis displacement and subsidence in the treatment of lumbosacral spinal tuberculosis treated by anterior titanium cage combined with lateral double screw- rod fixation at the end of follow-up, this may be due to the short follow-up period.

In this study, the maximum Von Mises stresses in group 3 were the lowest among the three groups in all six directions, indicating that a titanium cage combined with posterior double screw-rod fixation could provide a greater stress reduction in the cortical bone around the screws than the other two internal fixation methods, thereby reducing the incidence of screw loosening after operation to a certain extent. Jiang et al. ^32^ also found that posterior screw-rod fixation in the treatment of lumbosacral tuberculosis of the spine can better reconstruct the stability of the spine. Bezer et al.^33^ also had similar results. The maximum Von Mises stresses in the cortical bone around the screws in group 1 were higher than those in group 2 in the FLX, LT and RT directions but lower than those in the EXT, LLB and RLB directions. The stresses in the cortical bone around the screws in group 1 and group 2 have different advantages in different directions of motion, among which the former has more advantages in the FLX, LT and RT directions, which helps prevent screw loosening.

The results of this study showed that the maximum Von Mises stress of the adjacent disc in group 1 (L3-4 disc) in the EXT direction was slightly higher than that in group 2 and group 3, indicating that titanium cage combined with lateral double screw-rods fixation might be more likely to cause the degeneration of the adjacent disc in the EXT. The maximum Von Mises stress analysis of facet joints showed that the maximum stress of the L3-4 facet joints in group 1was slightly higher than that in group 2 and group 3 in the direction of LLB and RLB, indicating that the titanium cage combined with lateral double screw-rods fixation was more likely to cause the degeneration of adjacent facet joints during lateral bending. The maximum Von Mises stresses of L4-5 and L5-S1 facet joints of group 3 in all directions were zero, which indicated that the titanium cage combined with posterior double screw- rod fixation can obtain strong stability, so that the facet joints of the operative site did not produce local stress. Thus, group 3 was more advantageous in reconstructing surgical site stability and preventing adjacent disc and facet joint degeneration.

This study has several limitations. In this study, FE modelling of the intact lumbosacral spine was based on CT data obtained from a young, healthy, male patient. However, these data might have neglected the influence of pathologies on the biomechanical performance of the lumbosacral spine. Second, in order to compare the biomechanical properties of the three surgical methods, we constructed the identical geometry of the lumbosacral spine. However, different surgical approaches differently affect spine lordosis and profile, which is not reflected in our study. Third, we adopted simplified loads to simulate the mechanical environment, which is somewhat different from the complex human body, and the research results still need to be further verified by in vivo studies. This FE analysis only constructed the lumbosacral segment tuberculosis model after L5 vertebral body resection but failed to cover the involvement of other vertebral bodies or multiple models. Hence, further studies are needed to analyse these specific situations.

## Conclusions

Titanium cage with posterior double screw-rod fixation has more advantages in reconstruction of lumbosacral spinal stability, prevention of screw loosening and alleviating degeneration of adjacent intervertebral discs and facet joints. Autologous bone fixation with posterior double screw-rod fixation has a higher risk of screw fracture and a lower incidence of prosthesis subsidence than the other two fixation methods. The titanium cage with the lateral double screw-rod fixation has better mechanical properties in some directions, but compared with the other two internal fixation methods do not show absolute advantages.

## Data Availability

The datasets used and/or analysed during the current study available from the corresponding author on reasonable request.
